# SnFe_2_O_4_ Nanozyme Based TME Improvement System for Anti-Cancer Combination Thermoradiotherapy

**DOI:** 10.3389/fonc.2021.768829

**Published:** 2021-10-20

**Authors:** Wen Zeng, Chunping Liu, Shuntao Wang, Ziqi Wang, Qinqin Huang

**Affiliations:** ^1^ Department of Molecular Pathology, The Second Affiliated Hospital of Zhengzhou University, Zhengzhou, China; ^2^ Department of Ophthalmology, Zhongnan Hospital of Wuhan University, Wuhan, China; ^3^ Department of Breast and Thyroid Surgery, Union Hospital, Tongji Medical College, Huazhong University of Science and Technology, Wuhan, China; ^4^ Key Laboratory of Artificial Micro- and Nano-Structures of Ministry of Education, School of Physics and Technology, Wuhan University, Wuhan, China

**Keywords:** radiotherapy, photothermal therapy, hydrogel, nanozymes, tumor therapy

## Abstract

High doses of radiotherapy (RT) are associated with resistance induction. Therefore, highly selective and controllable radiosensitizers are urgently needed. To address this issue, we developed a tin ferrite (SFO)-based tumor microenvironment (TME)-improved system (SIS) that can be used in combination with low-dose radiation. The SIS was delivered *via* intratumoral injection directly to the tumor site, where it was stored as a ration depot. Due to the photothermal properties of SFO, SIS steadily dissolved under near-infrared (NIR) laser irradiation. Simultaneously, the dual glutathione oxidase (GSH-OXD) and catalase (CAT) activities of the SFO nanozyme significantly lowered the content of GSH in tumor tissues and efficiently catalyzed the conversion of intracellular hydrogen peroxide to produce a large amount of oxygen (O_2_) for intracellular redox homeostasis disruption, thus reducing radiotherapy resistance. Our *in vivo* and *in vitro* studies suggested that combining the SIS and NIR irradiation with RT (2Gy) significantly reduced tumor proliferation without side effects such as inflammation. To conclude, this study revealed that SFO-based nanozymes show great promise as a catalytic, radiosensitizing anti-tumor therapy.

## Introduction

Cancer, which can strike at any age and affect anybody, remains a serious threat to human life and health in today’s society, despite current scientific advancements (1–3). Radiotherapy (RT), either alone or in combination with other cutting-edge treatments, is widely utilized to treat cancer patients (4). RT is based on the use of high-energy X-rays or gamma rays to generate radiation-induced DNA damage and triggers the development of large amounts of harmful reactive oxygen species (ROS) (4). Both radiation-induced DNA damage and ROS production that exceeds the potential of the cell to neutralize these free radicals result in cell death by apoptosis reducing the size of the tumor (5, 6). However, while RT kills tumor cells, it does so at the expense of nearby cells and tissues in the human body. Radiation produces ROS in a dose-dependent manner, resulting in better treatment outcomes at higher doses (7, 8). However, high-dose radiotherapy might cause systemic effects, including fatigue, loss of appetite, bone marrow suppression, radiotherapy-induced secondary and primary malignancies, and infertility, as well as local radiation damage. Local liver injury can lead to altered activity or even liver failure in more serious cases (9, 10). Furthermore, while RT has some efficacy, the tumor microenvironment (TME) in solid tumors frequently exhibits high levels of glutathione oxidase (GSH-OXD) expression, since GSH plays an essential role in anti-tumor radiation *via* GSH spontaneous reaction or GSH S-transferase catalyzed reaction with xenoorganisms (11–13). Moreover, since GSH is a reducing agent it can directly eliminate ROS which reduces the effectiveness of ROS-based therapies (14). Therefore, a decrease in cellular GSH content can effectively promote radiation sensitization and, thus, improve RT efficacy (15, 16). The employment of alternative catalysts to decrease the levels of GSH is expected to have a good synergistic impact when paired with irradiation, allowing a reduction of the dose of radiation without compromising the therapeutic effect.

Since there are various pathways of GSH metabolism and various types of chemical reactions involving GSH, its elimination can be accomplished in a variety of ways (17, 18). Converting GSH to its oxidized state through direct interactions has become one of the most commonly used methods for lowering GSH levels (11, 13, 19). For example, Bao et al. achieved radiation sensitization by creating a composite nanomaterial containing MnO_2_ to improve tumor hypoxia and lower intracellular GSH levels (20). Qu et al. developed MoS_2_@AIBI-PCM, a composite nanomaterial in which GSH oxidation is effectively achieved through reaction with MoS_2_ without releasing hazardous metal ions, resulting in significant tumor death and good biocompatibility during therapy (21). Tin ferrite (SnFe_2_O_4_, abbreviated as SFO) is a novel nanomaterial that stimulates both GSH-OXD and high-activity CAT (22). SFO can reduce GSH and act as a catalyst for the conversion of H_2_O_2_ to O_2_ to produce sufficient O_2_ to sensitize the TME, and is expected to act synergistically with RT. These nanomaterials can reach tumor tissues *via* blood circulation upon intravenous injection. Despite the fact that these nanomaterials have proven effective against the GSH system they are vulnerable to the activity of the immune system and are easily cleared from the bloodstream by the liver and kidneys, which considerably reduces their anti-tumor efficiency. Various drug delivery systems such as liposomes have been designed and developed in recent years that represent safer and more effective cancer treatments (17, 23). However, the interference of a series of *in vivo* biological barriers, including the blood circulation, vascular extravasation, accumulation at the tumor location, tumor depth of stromal infiltration, and tumor cell internalization, have limited intracellular drug release.

Traditional drug delivery systems are susceptible to issues such as poor drug loading, complicated synthesis methods, early drug leakage or slow-release, and the long-term toxicity brought on by the carrier’s presence in the body over an extended period (24, 25). Recently, light-responsive hydrogels with minimum invasiveness have received a lot of attention as a controlled drug release platform (26–28). The hydrogel progressively solidifies after being injected into tumor tissue and can serve as a rationing depot over a long period (29). After one injection, this form of local administration can be used repeatedly. Furthermore, parameters such as laser power and irradiation period can be modified to alter the medication release rate, extending the applicability of this treatment method. Recently, Zhu et al. were the first to employ an agarose hydrogel to deliver anti-tumor aggregation-induced emission-based luminogens (AIEgen) material. As a photothermal agent, Prussian blue (PB) nanozyme stimulates the disintegration of the hydrogel while also stimulating CAT to scavenge H_2_O_2_ to sensitize the tumor microenvironment (30). Following that, under the irradiation of low-power white light, AIEgens can produce ROS under sufficient oxygen levels to promote tumor ablation. Zhang et al. developed a black phosphorus-based injectable hydrogel for photothermal therapy, which employs external photoexcitation to release cancer drugs, resulting in accurate and safe cancer treatment (31). In addition, thermal-radiotherapy is a combined treatment mode. Photothermal therapy can not only kill tumors alone, but also inactivate cells, thus sensitizing radiotherapy (32). In view of these findings, we hypothesized that delivering SFO to the TME using hydrogels would enhance the efficacy of low-dose radiation.

**Scheme 1:**
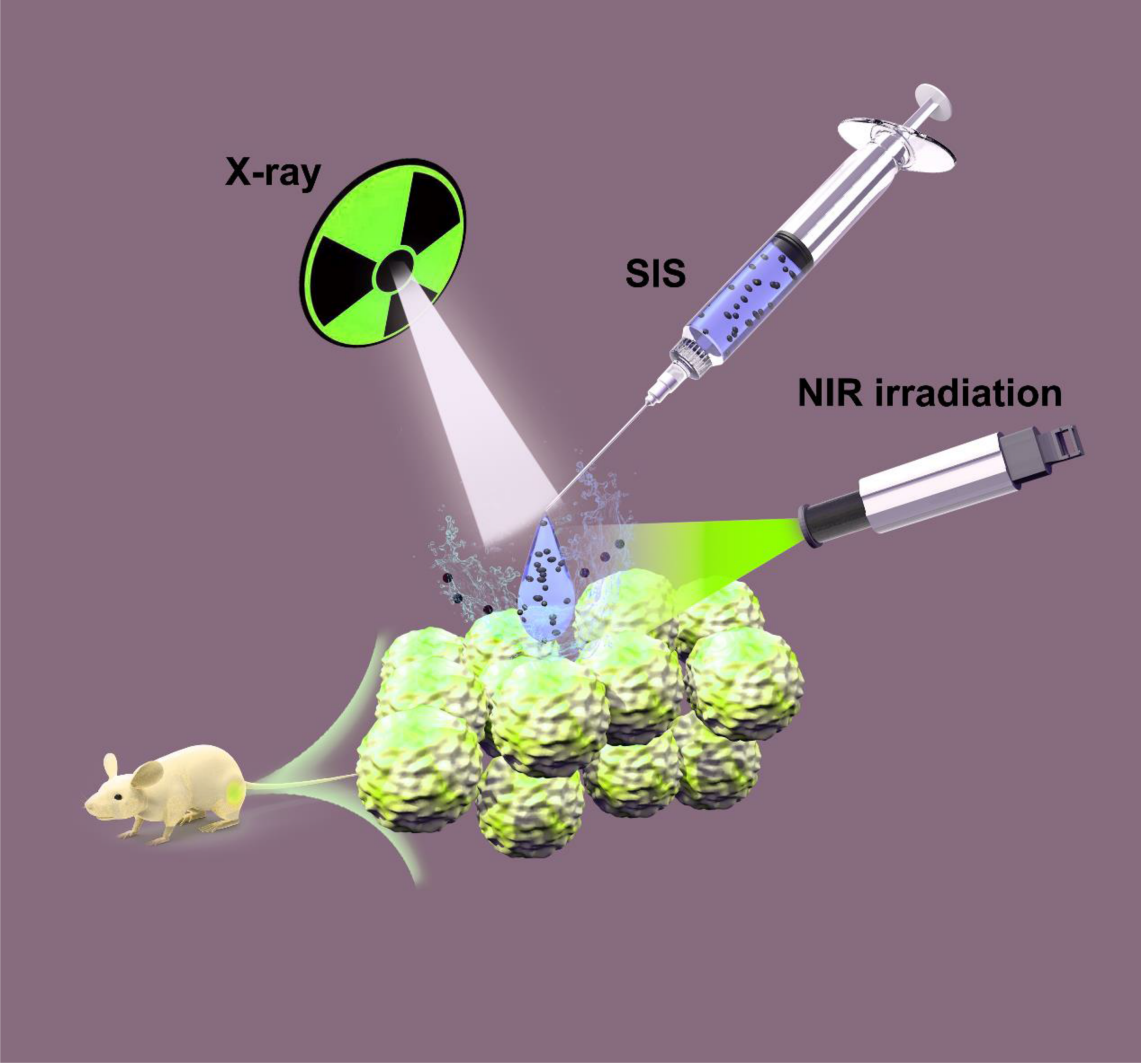
SIS system was used for anti-cancer combination thermoradiotherapy.

In this study, we developed a method using intratumoral delivery of an injectable hydrogel containing SFO nanoparticles for combined photothermal and radiotherapy (**Scheme 1**). Agarose hydrogels have been approved by the US Food and Drug Administration (FDA) owing to their reliable biosafety. Therefore, we prepared an SFO-based TME-improved system (SIS) by loading SFO nanoparticles into an agarose hydrogel. SFO nanoparticles serve as a photothermal agent (PTA) in this system due to their outstanding photothermal performance. SFO turns light energy into heat energy when an 808 nm near-infrared (NIR) laser irradiates the SIS system, causing the temperature of the agarose hydrogel to rise and reversible hydrolysis and softening to occur. When SIS diffuses into the local TME, the SFO nanozyme lowers intracellular GSH levels and simultaneously catalyzes the conversion of H_2_O_2_ to O_2_, due to its stimulation of GSH-OXD and CAT activities, respectively, increasing the sensitivity of the TME to radiation. The SIS functions as an SFO storage control, to achieve regulated drug release following its intratumoral injection of local malignancies. Our *in vivo* and *in vitro* experiments showed that SIS is effective in treating tumors without any off-target toxicity. In conclusion, the SIS nanosystem has a wide range of clinical potential in synergetic anti-tumor therapy.

## Results and Discussion

**Figure 1:**
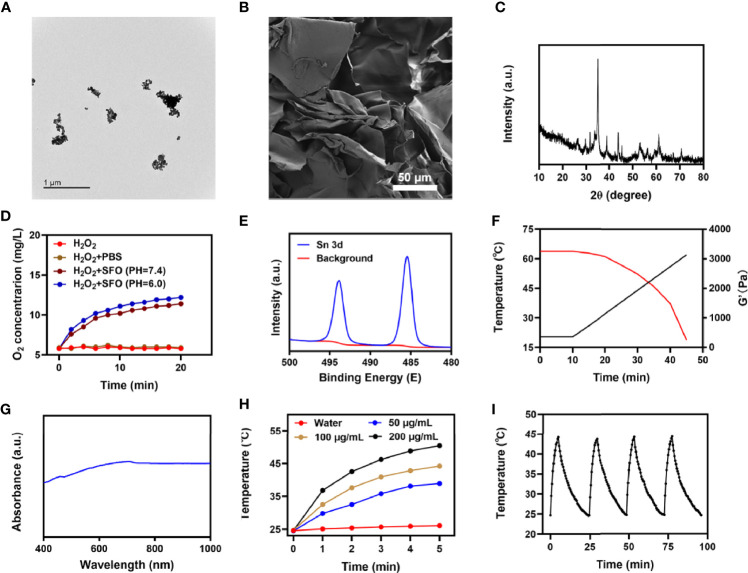
**(A)** TEM image of SFO. **(B)** SEM image of hydrogel. **(C)** XRD pattern of SFO. **(D)** O_2_ generation in H_2_O_2_ solution added with PBS or SFO under different pH values (7.4 and 6.0). **(E)** Sn 3d spectrum of XPS spectra of fresh SFO. **(F)** Rheological and temperature curves (red and black, respectively) for the prepared SIS in response to 0.5 W/cm^2^ 808 nm laser irradiation. **(G)** SFO absorbance spectra. **(H)** Heating curves for the different concentrations of SFO nanoparticles solutions upon laser irradiation at 808 nm (0.5 W/cm^2^) for 5 min. **(I)** Temperature variation of an SFO solution at 200 μg/mL under cyclic laser irradiation.

We first obtained a transmission electron microscope (TEM) image of SFO, as shown in **Figure 1A**. SFO has outstanding dispersibility and small size with an average of 16.4 ± 1.4 nm. Since nanomaterials are easily cleared by the kidney it is difficult to achieve the desired therapeutic effect by methods that require circulation in the bloodstream (33). Therefore, we used a hydrogel delivery technique that considerably improved the applicability of SFO. The hydrogel was prepared using a basic hydrothermal technique and analyzed by scanning electron microscopy (SEM). As shown in **Figure 1B**, the SEM images reveal the complex pore structure of the hydrogel. The results of X-ray diffraction (XRD) and X-ray photoelectron spectroscopy (XPS) are shown in **Figures 1C, E**, respectively. The ability of SFO nanozymes to produce O_2_ from hydrogen peroxide is essential for the treatment of hypoxic tumors. **Figure 1D** shows that SFO nanozyme interacts with H_2_O_2_ and effectively produces O_2_. The rheological values of SIS were measured at various temperatures (**Figure 1F**). The results revealed that as the temperature rises SIS gradually dissolves, accompanied by a gradual decrease in storage modulus. This is in line with the hydrogel’s rheological properties. One of the most essential factors for evaluating PTA is photothermal stability (34, 35). A powerful photothermal treatment can be assisted by a good photothermal agent. **Figure 1G** shows that SFO has a wide absorption region (650-900 nm). To test the photothermal performance of SFO nanoparticles, solutions were prepared with different concentrations of SFO (0, 50, 100, 200 μg/mL). **Figure 1H** shows that, assuming all other parameters remain constant, the heating impact of the solution increases as the SFO concentration rises. At 100 μg/mL SFO, the temperature increased by roughly 19.5°C after 5 min of laser irradiation. Next, a 200 μg/mL SFO solution was repeatedly heated for 5 min using an 808 nm NIR laser and allowed to cool to ambient temperature (**Figure 1I**). The heating curves of each cycle were identical and the variations in peak temperatures were minor, demonstrating that the photothermal conversion capability of the SFO nanoparticles was stable and reproducible over 4 repeated heating and cooling cycles. These findings suggested that the SFO nanoparticles had good photothermal stability. Furthermore, the photothermal conversion efficiency (η) of the SFO was calculated from the data of **Figure 1I** and was found to be 38.5%, which was greater than various other materials such as Au nanostars (36.4%) and Ti3C2 nanosheets (30.6%) (36, 37).

**Figure 2:**
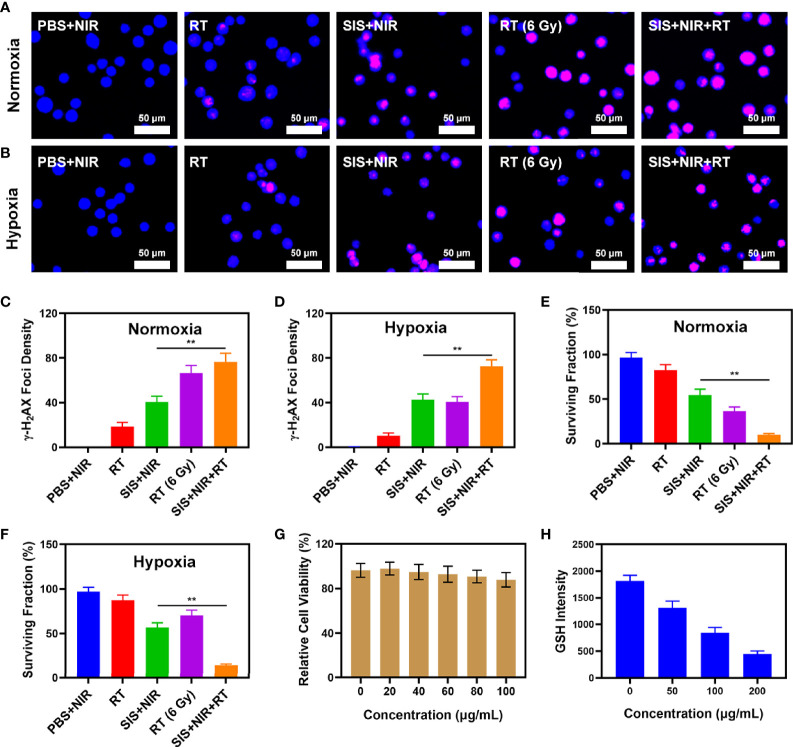
**(A)** The CLSM images of 4T1 cells under different treatment in normoxia condition. The nuclei were stained with DAPI (blue) and DSBs were stained with γ-H_2_AX (red). **(B)** The CLSM images of 4T1 cells under different treatment in hypoxia condition. The nuclei were stained with DAPI (blue) and DSBs were stained with γ-H_2_AX (red). **(C)** The density of γ-H_2_AX foci in **(A)** was determined based on analyses of 100 cells per treatment group (γ-H_2_AX foci/100 μm^2^, n = 3). **(D)** The density of γ-H_2_AX foci in **(B)** was determined based on analyses of 100 cells per treatment group (γ-H_2_AX foci/100 μm^2^, n = 3). **(E)** Colony formation assays were conducted using 4T1 cells treated with radiation under normoxia condition (n = 3). **(F)** Colony formation assays were conducted using 4T1 cells treated with radiation under hypoxia condition (n = 3). **(G)** Dark cytotoxicity of SFO on 4T1 cells. **(H)** Quantitative analysis of GSH levels for different SFO concentration. **P < 0.01; Student’s t-test.

The SIS system is well-structured and performance oriented. Anti-tumor experiments *in vitro* are presently underway. While SFO has the potential to disrupt the ecological balance of cancer cells and hence increase the efficacy of radiation, it can do so only when it is present in tumor tissue. The immune system may recognize stimuli from a variety of foreign invaders and part of that stimulation may trigger the immune response, resulting in immunity, while other stimuli may result in tolerance. When tumor cells are exposed to radiation, double-stranded DNA breaks (DSB) occur at certain sites, providing insight into radiation sensitization (38). Measuring the fluorescence intensity of the histone variant H_2_AX phosphorylated is a specific and sensitive technique to detect DSB formation following DNA damage (39). Therefore, we analyzed the density of H2AX foci in the nuclei of 4T1 cells after various treatments, including 1) PBS + NIR; 2) RT (2Gy); 3) SIS + NIR; 4) High dose RT (6Gy); and 5) SIS + NIR + RT, both under normoxic and hypoxic conditions. Under normoxic conditions, 2 Gy of radiation caused substantial DNA damage and when the dose was raised to 6Gy, the DSB effect increased. However, in hypoxic cells, the effect was not satisfactory. The DSB effect in the 6 Gy RT group under hypoxia conditions was only about 40.6%. It is important to mention that 808 nm laser irradiation combined with SIS achieved about 40% γ-H2AX formation whether in hypoxia or normoxia conditions. Notably, SIS + NIR + RT displayed the strongest effect, up to 76.4% and 72.6% γ-H2AX foci development in normoxic and hypoxic conditions, respectively, which was greater than the 6 Gy RT groups (**Figures 2A**–**D**). The uniform and significant differences between each experimental group were linked to the synergistic effect of SIS as a photothermal agent in PTT, SFO as CAT and GSH-OXD, and sensitization to low-dose radiation. Colony formation assays also showed that the SIS + NIR + RT group demonstrated considerable tumor growth inhibition compared with controls under both normoxic and hypoxic conditions (**Figures 2E, F**, respectively). SFO was added to 4T1 cells at various doses (0, 5, 10, 20, 40 μg/mL) and after 24 hours of incubation cell viability was assessed (**Figure 2G**). Even at high concentrations, cell viability did not decline significantly. Therefore, our results show that SFO NPs exhibit high biocompatibility. GSH is a rich endogenous antioxidant that can maintain cellular redox balance and inhibit cell damage caused by ROS (40). Therefore, we studied the ability of SFO nanozymes to deplete GSH. With increasing SFO concentrations, GSH depletion increased significantly (**Figure 2H**). Taken together, these findings motivated us to fully investigate the anti-tumor efficacy of SFO *in vivo*.

**Figure 3:**
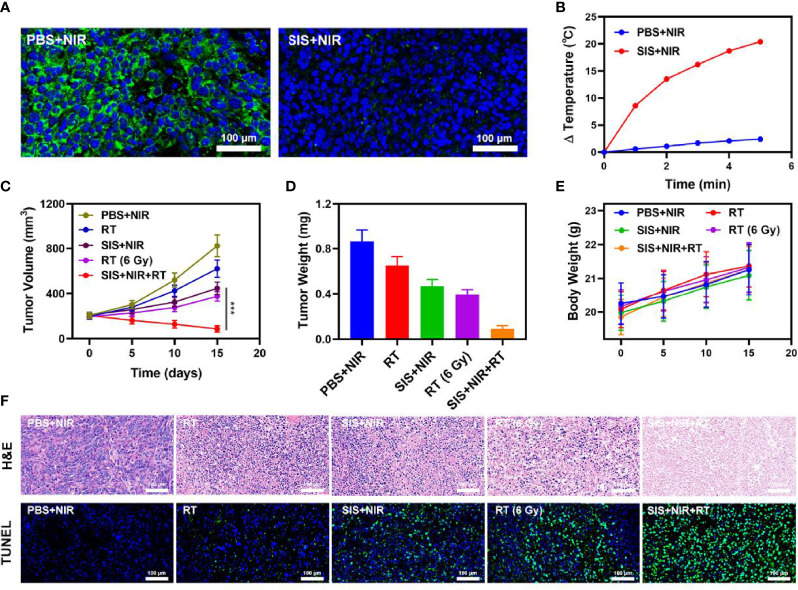
**(A)** Representative images of tumor tissue sections stained with anti-PIMO (green) and DAPI (blue) following the indicated treatments. **(B)** Temperature increases in mice implanted with 4T1 tumors following 808 nm laser irradiation (0.5 W/cm2) for 5 min in the indicated treatment groups. **(C)** Tumor volume change over time in groups treated as indicated. **(D)** Average tumor weight values associated with the indicated treatments. **(E)** Changes in body weight in response to the indicated treatments. **(F)** H&E and TUNEL stained tumor sections from the indicated treatment groups. ***P < 0.005; Student’s t-test.

**Figure 4:**
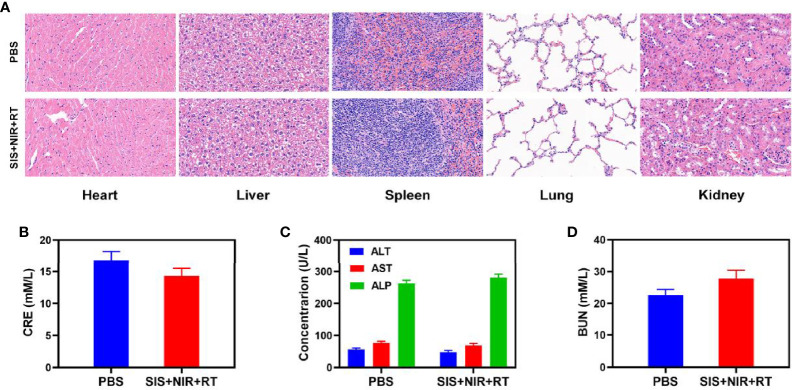
Result of *in vivo* safety experiments. **(A)** Histopathological analysis results (H&E stained images) of the major organs, heart, lung, liver, kidneys, and spleen, of mice that were exposed to different treatments 16 days post-injection. Blood biochemistry data including kidney function markers: **(B)** liver function markers: CRE, **(C)** ALT, ALP, and AST and **(D)** BUN after various treatments.

The proliferation of malignant tumors is associated with local intratumoral hypoxia and, as a result, cancerous tissues are less susceptible to the lethal DNA damaging effects of ionizing radiation than aerobic cells, reducing the sensitivity and increasing the resistance towards radiotherapy (41). We explored the effect of SIS on the oxygen content of the tumor. The results showed that SIS + NIR reduced the hypoxic staining (PIMO-positive cells) in the tumor (**Figure 3A**), while the control group (PBS + NIR) showed obvious hypoxic areas, confirming that the SFO actively catalyzes the conversion of local H_2_O_2_, producing a large amount of oxygen to relieve the tumor hypoxia. In view of its good *in vitro* performance as a PTA and radiosensitizer, we next investigated the photothermal conversion impact of SFO *in vivo*. BALB/c mice were injected subcutaneously with 4T1 cells to develop tumors. **Figure 3B** shows the temperature change curves for the PBS and SIS groups after 10 minutes of 808 nm NIR laser irradiation at 0.5 W/cm^2^. Within 10 minutes of receiving SIS, the temperature increased about 20.4°C, whereas the PBS group experienced only little increase in temperature. Tumor tissues have lower heat resistance than normal cells, resulting in tumor cells being selectively destroyed at high temperatures (42–47°C). The efficacy of SIS-mediated anti-tumor activity was then tested in mice bearing 4T1 tumors. To investigate the primary effect of the SIS, BALB/c mice were subcutaneous injected into the right flank with 1 × 10^6^ 4T1 cells. When the primary tumor volumes reached 200 mm^3^, the mice were randomly divided into 5 groups (each group included 5 mice): 1) PBS + NIR; 2) RT (2Gy); 3) SIS + NIR; 4) High dose RT (6Gy); 5) SIS + NIR + RT. The SFO concentration was 1 mg/kg in groups 3, and 5. The mice received therapy every five days for 16 days. The tumor volumes of the PBS + NIR group and the low dosage RT treated group increased rapidly over the 2 weeks of treatment, as illustrated in **Figure 3C**. The SIS + NIR treatment had a tumor-suppressing impact that was moderate. Following intratumoral injection of SIS, the hydrogel will disintegrate once subjected to laser radiation and release SFO. SFO then catalyzes the intratumoral conversion of H_2_O_2_ to produce O_2_ in situ, which increases radiation sensitization. Furthermore, SFO decreases GSH levels in the tumors, further amplifying the radiation effect. The SIS + NIR + RT treatment, which included SFO, had the most potent therapeutic impact, with growth curves of tumor volume nearly completely suppressed during therapy. As the combination of 808 nm laser irradiation and RT could play the role of mutual promotion, SFO could simultaneously enhance RT and PTT. The tumor mass of the mice was also in agreement with the volume curve (**Figure 3D**). No weight changes were observed in the treatment group throughout the study, indicating that the treatment did not cause any significant systemic toxicity in the mice (**Figure 3E**), which is noteworthy because many treatments are associated with severe systemic toxicity, which severely hampers future medical applications of the material (30). We obtained slices of tumor tissue for staining. Hematyloxin and eosin (H&E) and TUNEL staining (**Figure 3F**) revealed that tumors from the SIS combined thermal radiation group had a significant percentage of cell necrosis. Furthermore, SFO activation did not result in systemic loss, as shown in **Figure 4**. Following the treatment, the vital organs (heart, liver, spleen, lungs, and kidney) were without any inflammation or damage. Moreover, the liver and kidney indexes were also normal. While many nanomaterials have high therapeutic efficacy, they also have a high risk of systemic toxicity, which limits their future clinical applications. Our *in vivo* results showed that our unique and powerful SIS-enhanced combination treatment not only achieved a high level of biological safety but also sensitized the TME, enhancing the efficacy of RT.

## Conclusion

In conclusion, by encapsulating SFO nanozymes in an agarose hydrogel, we developed an injectable light-controlled SFO-based hydrogel as a TME-sensitizing system named SIS. The nano-system allowed the combination of low-dose radiation with other therapies greatly improving tumor treatment outcomes. SFO nanoparticles are outstanding radiosensitizers and PTAs due to the nanozyme catalysis and superior photothermal effect in the NIR-I region. The agarose hydrogel underwent regulated and reversible hydrolysis and a softening under the NIR laser power, resulting in the light-triggered release of SFO nanoparticles and hydrogel deterioration. The release rate of SFO nanoparticles is adjustable by changing the parameters. More importantly, by injecting the hydrogel intratumorally, the concentration of SFO nanoparticles in tumor tissues will be significantly raised, a single injection allowing for multiple treatments *in vivo*. It is worth emphasizing that after photothermal treatment, we observed an increase in of O_2_ content of the tumor cells, which considerably boosted radiotherapy efficacy. The SIS exhibits outstanding cancer cell killing efficacy and tumor ablation properties in both *in vitro* and *in vivo* tests, with good stability and biocompatibility, and low toxicity. In conclusion, SIS has great potential in anti-cancer combination therapy.

## Data Availability Statement

The original contributions presented in the study are included in the article/**Supplementary Material**. Further inquiries can be directed to the corresponding author.

## Ethics Statement

The animal study was reviewed and approved by Administrative Committee on Animal Research of the Zhengzhou University.

## Author Contributions

Conceived and designed the experiments: ZW, WZ, QH, and SW. Performed the experiments: ZW and CL. Contributed reagents/materials/analysis tools: ZW and WZ. Revised the polished the article: QH. All authors contributed to the article and approved the submitted version.

## Funding

This work was supported by the National Natural Science Foundation of China (31800085).

## Conflict of Interest

The authors declare that the research was conducted in the absence of any commercial or financial relationships that could be construed as a potential conflict of interest.

## Publisher’s Note

All claims expressed in this article are solely those of the authors and do not necessarily represent those of their affiliated organizations, or those of the publisher, the editors and the reviewers. Any product that may be evaluated in this article, or claim that may be made by its manufacturer, is not guaranteed or endorsed by the publisher.
