# Supplementary material for: SnFe2O4 Nanozyme Based TME Improvement System for Anti-Cancer Combination Thermoradiotherapy
Source: Front Oncol. 2021 Oct 20;11:768829. doi: 10.3389/fonc.2021.768829 (PMC8564484; doi:10.3389/fonc.2021.768829)
Supplement: Supplementary file 1 [file DataSheet_1.docx]

Experimental Procedures

**Materials and reagents.**

Iron (II) chloride tetrahydrate (FeCl_2_·4H2O), Tin chloride dihydrate (SnCl_2_·2H2O), NaOH, ethylene glycol and ammonium hydroxide solution were purchased from Sinopharm Chemical Reagent Co., Ltd. Agarose was purchased from Yare Shanghai. The reduced GSH assay kit was purchased from Nanjing Jiancheng Bioengineering Institute. The other reagents used in this work were purchased from Sinopharm Chemical Reagent (China) and Aladdin-Reagent (China).

**Cell culture**

4T1 mouse breast cancer cell line was obtained from the Cell Bank of the Chinese Academy of Sciences and incubated in RPMI-1640 medium supplemented with 10% FBS in a humidified atmosphere at 37℃. Cell cultures under normoxic conditions (pO_2_: 21%) were maintained in a humidified incubator at 37℃ in 5% CO_2_ and 95% air. Hypoxic conditions (pO_2_: 2%) were produced by placing cells in a hypoxic incubator (Moriguchi, Japan) in a mixture of 2% O_2_, 5% CO_2_, and 93% N_2_.

**Synthesis of SFO**

Solvothermal method was adopted to synthesize the magnetic SnFe_2_O_4_ nanoparticles. Firstly, SnCl_2_·2H_2_O (0.017 M) and FeCl_2_ • 4H_2_O (0.034 M) were dispersed in ethylene glycol (25 mL) under magnetic stirring to obtain a mixture solution. Then, NH_4_OH solution (5 mL) was added and adjusted with NaOH to make sure that the pH of the solution was around 10. Soon afterward, the solution was transferred into autoclave and heated to 200 °C for 15 h. After cooling down to room temperature, the samples were obtained by centrifugation, and rinsed with ethanol and deionized water alternately several times, which is abbreviated as SFO. The morphology structures of SFO were observed by the TEM (JEOL-2100). UV-vis spectra of different samples were recorded by the UV-vis spectrophotometry Lambda 35 (Perkin-Elmer). XPS spectra were recorded by ESCAlab250 (Thermal Scientific). XRD was measured on a D8 Advance (Bruker- AXS, Germany).

**Preparation and characterization of SFO nanoparticles based TME improvement system (SIS)**

The general protocol for the hydrogel preparation is as follows. The prepared SFO (10 mg/mL in PBS) was added into 2% agarose solution to form SIS. Scanning electron microscopy (SEM) images were captured on a Hitachi FE-SEM S4800 instrument with an acceleration voltage of 3 kV.

**Rheological Test**

Rheology experiments were performed on an Anton Paar rheometer. Hydrogel samples of different temperatures were prepared and gently placed on the middle of a 15 mm diameter parallel plate with a proper gap. Dynamic oscillatory frequency sweep measurements were conducted at a 1% strain amplitude. To prevent the evaporation of water, a lid was prepared on the top.

**Photothermal Conversion Efficiency(**[**1**](#_ENREF_1)**)**

A 808 nm NIR laser (Changchun New Industries Tech.Co., Ltd., Changchun, China) with irradiation powers was used to stimulate the concentrations of SFO (200 ug/mL) in an aqueous medium. The photothermal images of the SFO-based suspensions during laser irradiation were recorded every 30 s using an infrared thermal imaging system. The NIR laser source was equipped with a 4 mm diameter laser module with an adjustable power. The photothermal conversion efficiency was calculated using the following equation:


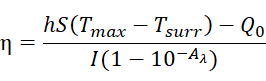


where h is the heat transfer coefficient, S is the surface of the container, T_max_ and T_surr_ are the equilibrium temperature and ambient temperature, respectively. Q_0_ is the heat associated with the light absorbance of the solvent, A_λ_ is the absorbance of SFO at 808 nm, and I is the laser power density. According to the above equation, the η value of SFO was determined to be about 38.5%.

**Evaluation the generation of oxygen**

PBS and SFO were suspended in 3% H_2_O_2_ solution or ultrapure water (8 mL), respectively. A DOG-3082 oxygen dissolving meter was used to monitor the real-time concentration of oxygen.

**γ-H_2_AX immunofluorescence analysis.**

4T1 cells were incubated at 37 °C for 24 h in five groups: 1) PBS+808 nm laser (NIR, 0.5 W/cm2); 2) radiotherapy (RT, 2Gy); 3) SIS + NIR; 4) RT (6Gy); 5) SIS+NIR+RT (2Gy). Then, cells in group 1, 3 and 5 were irradiated with the NIR. After 2 h, group 2, 4 and 5 were treated by RT. The dose of group 2 and 5 were 2 Gy. The dose of group 4 was 6 Gy. The dose of SFO is 200 μg/ml. After three-times rinse with PBS, cells were fixed in 4% paraformaldehyde for 20 min, treated by 0.1% Triton-X-100 and later blocked at room temperature for 2 h. Washed with PBS for three times, the cells were dyed with DAPI and secondary anti-γ H2AX antibody with 5% FBS and 1% Trition-X-100 before washed with PBS. The cells immunofluorescence was analyzed by a fluorescent microscope (IX81, Olympus, Japan). A similar experiment was performed under hypoxic conditions, but the cell culture conditions were changed to hypoxic.

**Clonogenic survival assay**

The effect of oxygen on the radiosensitivity of 4T1 cells was assessed by a clonogenic assay. 500 cells per flask were seeded in 25 cm^2^ flasks and cultured in normoxia for 24 h. Flasks were treated under following conditions: 1) PBS+808 nm laser (NIR, 0.5 W/cm^2^); 2) radiotherapy (RT, 2Gy) ；3) SIS+NIR；4) RT (6Gy) ；5) SIS+NIR+RT (2Gy). The dose of SFO is 200 μg/ml. The cells were washed with PBS, and then exposed to 2 or 6 Gy of radiation in sealed flasks containing 5 mL of complete medium. To allow formation of colonies, after radiation, the cells were then incubated for another 10 days, without changing the media. To determine the clonogenic survival rate, cultures were first fixed with paraformaldehyde, and then stained with trypan blue. Colonies with greater than 50 cells were counted under the microscope, and the survival fractions (SF) were calculated using the formula SF = colonies counted/cells seeded.([2](#_ENREF_2)) A similar experiment was performed under hypoxic conditions, but the cell culture conditions were changed to hypoxic.

**Detection of Intracellular GSH.**

The commercially available GSH assay kit was used to detect the depletion of GSH. 4T1 cells were seeded in culture bottles for 24 h (37 °C, 5% CO2). Then, a SFO solution (0, 0.05, 0.1 and 0.2 mg/mL in medium) was added and incubated for 24 h. After that, each culture bottles were rinsed with PBS and centrifuged at 3000 rpm to collect 4T1 cells. Then, the 4T1 cells were suspended in 1 mL of PBS and processed by an ultrasound cell crusher. After that, 0.5 mL of the above 4T1 cells was added to 2 mL of regent one in an assay kit and centrifuged at 3500 rpm for 10 min. The depletion of intracellular GSH was measured by UV−vis spectroscopy.

***In vitro* biocompatibility of SFO**

The biocompatibility was measured by MTT assay. 4T1 cells were seeded in 96-well plates at a density of 5 × 10^3^ cells per well and incubated for 24 h. Afterwards, 4T1 cells were incubated with different concentration of SFO PBS solution. At the end of the incubation, 5 mg/mL MTT solution was added, and the plate was incubated for another 4 h. Finally, the absorbance values of the cells were determined by using a microplate reader (Emax Precision, USA) at 570 nm. The background absorbance of the well plate was measured and subtracted. The cytotoxicity was calculated by dividing the optical density (OD) values of treated groups (T) by the OD values of the control (C) (T/C × 100%).([3-5](#_ENREF_3))

**Animal tumor models**

Female BALB/c nude mice aged 4-5 week were purchased from Vital River Company (Beijing, China). 100 μL of 4T1 cell suspension (1×10^6^ cells per mL) were subcutaneous injected into each mouse to establish the tumor models. The animal experiments were carried out according to the protocol approved by the Ministry of Health in People’s Republic of PR China and were approved by the Administrative Committee on Animal Research of the Zhengzhou University.

***In vivo* hypoxia evaluation**

After the tumor size reached 200 mm^3^, the mice were divided randomly into 2 groups (n =3 per group): (1) PBS+NIR; (2) SIS+NIR. The dose of SFO is 1 mg/kg. The injection method is intratumoral injection. After 1h injection, the tumor sites of mice were irradiated by a 808 nm laser (0.5 W/cm^2^) for 10 min. A total of 30 min later, the mice were all sacrificed, and the tumors should be effectively stained with PIMO. The subsequent treatment of PIMO tumor components was achieved through MATLAB software.

***In vivo* infrared thermography**

To monitor the *in vivo* photothermal effect, SIS (SFO: 1 mg/kg) was intratumorally injected into the tumor-bearing mice, and then the tumors suffered from 0.5 W/cm^2^ irradiation for 5min at 1 h post-injection. PBS injection used as control group. Meanwhile, the temperature at the tumor was monitored using an infrared camera (Fotric 225).

***In vivo* antitumor study**

The mice were firstly divided randomly into 6 groups (each group included 5 mice): 1) PBS+808 nm laser (NIR, 0.5 W/cm2); 2) radiotherapy (RT, 2Gy); 3) SIS+NIR; 4) RT (6Gy); 5) SIS+NIR+RT (2Gy). The injection method is intratumoral injection. Among them, the dose of SFO in groups 3 and 5 are 1 mg/kg. NIR was conducted 1h after the injection. Mice body weight and tumor volume in all groups were monitored every 5 days. A caliper was employed to measure the tumor length and tumor width and the tumor volume was calculated according to following formula. Tumor volume = tumor length × tumor width^2^ / 2. After 15 days treatment, mice were sacrificed. Five main organs (heart, liver, spleen, lung and kidney) of all mice were harvested, washed with PBS, and fixed with paraformaldehyde for histology analysis. The blood samples from these mice (≈1 mL) were collected for blood biochemistry analysis. And the tumor tissues were weighed, and fixed in 4% neutral buffered formalin, processed routinely into paraffin, and sectioned at 4 μm. Then the sections were stained with hematoxylin and eosin (H&E) and TUNEL and finally examined by using an optical microscope (BX51, Olympus, Japan).

**Statistical analysis**

Data analyses were conducted using the GraphPad Prism 5.0 software. Significance between every two groups was calculated by the Student’s t-test. *P < 0.05, **P < 0.01, ***P < 0.005.

**References**

1. D. Zhu, M. Lyu, Q. Huang, M. Suo, Y. Liu, W. Jiang, Y. Duo and K. Fan: Stellate Plasmonic Exosomes for Penetrative Targeting Tumor NIR-II Thermo-Radiotherapy. *ACS Appl. Mater. Interfaces*, 12(33), 36928-36937 (2020) doi:10.1021/acsami.0c09969

2. D. Zhu, M. Lyu, W. Jiang, M. Suo, Q. Huang and K. Li: A biomimetic nanozyme/camptothecin hybrid system for synergistically enhanced radiotherapy. *J Mater Chem B*, 8, 5312-5319 (2020) doi:10.1039/d0tb00676a

3. D. Zhu, Y. Duo, S. Meng, Y. Zhao, L. Xia, Z. Zheng, Y. Li and B. Z. Tang: Tumor-Exocytosed Exosome/Aggregation-Induced Emission Luminogen Hybrid Nanovesicles Facilitate Efficient Tumor Penetration and Photodynamic Therapy. *Angew. Chem., Int. Ed.*, 59, 2-10 (2020) doi:10.1002/anie.202003672

10.1002/ange.202003672

4. D. Zhu, Z. Zheng, G. Luo, M. Suo, X. Li, Y. Duo and B. Z. Tang: Single injection and multiple treatments: An injectable nanozyme hydrogel as AIEgen reservoir and release controller for efficient tumor therapy. *Nano Today*, 37, 101091 (2021) doi:10.1016/j.nantod.2021.101091

5. D. Zhu, J. Zhang, G. Luo, Y. Duo and B. Z. Tang: Bright Bacterium for Hypoxia‐Tolerant Photodynamic Therapy Against Orthotopic Colon Tumors by an Interventional Method. *Adv. Sci.*, 2004769 (2021) doi:10.1002/advs.202004769
